# Patterns and regulation of ribosomal RNA transcription in *Borrelia burgdorferi*

**DOI:** 10.1186/1471-2180-11-17

**Published:** 2011-01-20

**Authors:** Julia V Bugrysheva, Henry P Godfrey, Ira Schwartz, Felipe C Cabello

**Affiliations:** 1 Department of Microbiology and Immunology, Basic Science Building, New York Medical College, Valhalla, NY 10595, USA; 2 Department of Pathology, Basic Science Building, New York Medical College, Valhalla, NY 10595, USA

## Abstract

**Background:**

*Borrelia burgdorferi *contains one 16S and two tandem sets of 23S-5S ribosomal (r) RNA genes whose patterns of transcription and regulation are unknown but are likely to be critical for survival and persistence in its hosts.

**Results:**

RT-PCR of *B. burgdorferi *N40 and B31 revealed three rRNA region transcripts: 16S rRNA-alanine transfer RNA (tRNA^Ala^); tRNA^Ile^; and both sets of 23S-5S rRNA. At 34°C, there were no differences in growth rate or in accumulation of total protein, DNA and RNA in B31 cultured in Barbour-Stoenner-Kelly (BSK)-H whether rabbit serum was present or not. At 23°C, B31 grew more slowly in serum-containing BSK-H than at 34°C. DNA per cell was higher in cells in exponential as compared to stationary phase at either temperature; protein per cell was similar at both temperatures in both phases. Similar amounts of rRNA were produced in exponential phase at both temperatures, and rRNA was down-regulated in stationary phase at either temperature. Interestingly, a *rel_Bbu _*deletion mutant unable to generate (p)ppGpp did not down-regulate rRNA at transition to stationary phase in serum-containing BSK-H at 34°C, similar to the relaxed phenotype of *E. coli relA *mutants.

**Conclusions:**

We conclude that rRNA transcription in *B. burgdorferi *is complex and regulated both by growth phase and by the stringent response but not by temperature-modulated growth rate.

## Background

*Borrelia burgdorferi*, the cause of Lyme disease, is maintained in nature in a sylvatic cycle that includes its arthropod host, *Ixodes scapularis*, and mammals such as deer and rodents [1,2]. The ability of *B. burgdorferi *to cycle successfully between different hosts, survive for prolonged periods of starvation in flat ticks and proliferate rapidly to reach sufficiently high numbers inside ticks taking a blood meal to permit transmission to mammals [1,3] suggests that *B. burgdorferi *may display novel and finely tuned mechanisms to regulate its growth in response to nutrient composition and other environmental cues [4-7]. Analysis of the genome of this bacterium, however, reveals a relative paucity of genes encoding regulatory molecules, suggesting that *B. burgdorferi *might control gene expression by ancillary methods such as growth rate-dependent control and the stringent response [8-10].

It is generally accepted that the nutritional quality of the environment acting through changes in bacterial growth rate regulates ribosome biosynthesis and ribosome availability. This regulation results in changes in ribosomal RNA (rRNA) concentration. rRNA molecules are thus the major factor regulating synthesis of ribosomes in *Escherichia coli *[11] and other prokaryotic and eukaryotic microorganisms [12]. *E. coli *has seven operons encoding rRNA genes; each operon contains genes for all three rRNA species which are transcribed as a single transcript and then processed into 16S, 23S and 5S rRNA [11,13]. This organization permits synthesis of equimolar amounts of each rRNA species. In *E. coli*, rRNA synthesis involves the transcription factor DksA [14]. It is negatively regulated by (p)ppGpp (guanosine-3'-diphosphate-5'-triphosphate and guanosine-3',5'-bisphosphate, collectively), a global regulator involved in bacterial adaptation to many environmental stresses, and positively regulated by the concentration of the initiating nucleoside triphosphates acting in trans on the P1 and P2 rRNA promoters [13]. The other major mechanism to control rRNA synthesis in *E. coli *is growth rate-dependent control [11]. Under this (p)ppGpp-independent control mechanism, ribosome concentration in each cell is proportional to growth rate.

**Figure 1:**
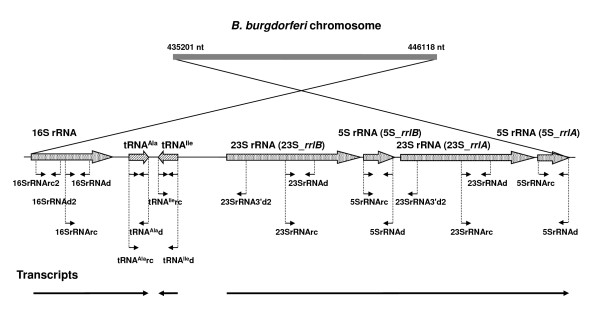
**Transcriptional organization of *B. burgdorferi *B31 chromosomal region containing rRNA genes **[10,15,16]. Short arrows indicate the position of primers from Table 1 used for analysis of rRNA expression in *B. burgdorferi*.

The *B. burgdorferi *chromosome contains a single 16S rRNA gene and two tandem sets of 23S and 5S rRNA genes located at nt435201-446118, as well as genes encoding transfer tRNAs for alanine (tRNA^Ala^) and isoleucine (tRNA^Ile^) [10,15,16] (Figure 1). All these genes except tRNA^Ile ^are present in the same orientation on the chromosome. Not only are patterns of transcription and regulation of rRNA genes uncharacterized in *B. burgdorferi*, but there is little information as to whether rRNA synthesis in this bacterium is regulated by the stringent response, by growth rate, or by some other mechanism. We previously found that *B. burgdorferi *N40 co-cultured with tick cells down-modulated its levels of (p)ppGpp and decreased *rel_Bbu _*expression while growing more slowly than in Barbour-Stoenner-Kelly (BSK)-H medium [17]. This simultaneous decrease in (p)ppGpp and growth rate was associated with down-modulation of 16S rRNA [18], and suggested that growth rate but not (p)ppGpp or the stringent response regulated rRNA levels in *B. burgdorferi*. A *B. burgdorferi *297 Δ*rel_Bbu _*deletion mutant lost both the ability to synthesize (p)ppGpp and to reach stationary phase cell densities as high as those of its wild-type parent even though the parent and the mutant multiplied at similar rates during exponential phase of growth [19].

We have now examined both the organization of transcription of *B. burgdorferi *rRNA and the influence of growth phase and the stringent response on rRNA synthesis. This information is especially critical to improving our understanding of the ability of *B. burgdorferi *to shift between the rapid growth of acute mammalian and arthropod infection and slow growth during persistence in these hosts [3,20,21]. Although the incompletely defined nutritional requirements of *B. burgdorferi *prevented experimental determination of whether *B. burgdorferi *rRNA synthesis was regulated by growth rate at a single temperature, we found that rRNA transcription was regulated by growth phase and that *rel_Bbu _*was required for down-regulation of rRNA at the entrance of *B. burgdorferi *to stationary phase.

## Results

### Transcription pattern of *B. burgdorferi *rRNA

**Figure 2:**
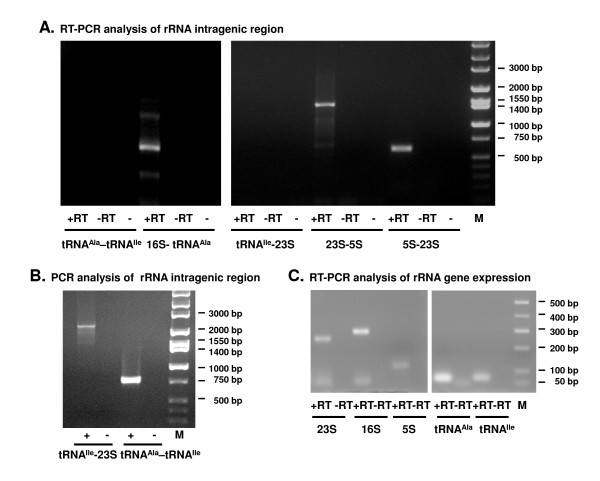
**Analysis of *B. burgdorferi *N40 rRNA gene transcription**. A. RT-PCR analysis of rRNA intragenic regions. +RT, complete reaction; -RT, reaction without reverse transcriptase; -, reaction without RNA. B. PCR analysis of rRNA intragenic regions. +, complete reaction; -, reaction without DNA. C. RT-PCR analysis of rRNA gene expression. +RT, complete reaction; -RT, reaction without reverse transcriptase.

RT-PCR analysis of the region coding for *B. burgdorferi *N40 rRNA using primers shown in Table 1 and Figure 1 demonstrated the presence of common transcripts (consistent with the expected 683 bp amplicon, Table 1) for 16S rRNA and tRNA^Ala^. The common transcripts detected for 23S and 5S rRNA (1403 bp) and for 5S and 23S-*rrlA *(631 bp) show that the 23S-5S-23S-5S region is expressed as a single transcript (Figure 2A). tRNA^Ile ^was transcribed independently of the upstream 16S rRNA and the downstream 23S-5S rRNA transcript since no amplicons were obtained with primers designed to amplify tRNA^Ala^-tRNA^Ile ^and tRNA^Ile^-23S rRNA segments (Figure 1, Figure 2A). However, PCR with these primers amplified products of the expected size (781 bp and 2522 bp, respectively) from genomic DNA (Figure 2B, Table 1). Transcripts consistent with expected sizes were also detected by RT-PCR for tRNA genes: tRNA^Ala ^(65 bp) and tRNA^Ile ^(69 bp) as well as for the three different rRNA genes: 23S, 248 bp; 16S, 288 bp; 5S, 112 bp (Figure 2C). Identical results were obtained with *B. burgdorferi *B31 (data not shown). These results confirm the prediction that the rRNA containing region in *B. burgdorferi *is transcribed as three independent transcripts [15,16].

**Table 1:** Oligonucleotide primers used in this study

Amplified gene/region	Primer Name	Sequence (5' → 3')	Amplicon (bp)
5S rRNA	5SrRNAd	CCCTGGCAATAACCTACTC	112
	5SrRNArc	CCCTGGTGGTTAAAGAAAAG	
16S rRNA	16SrRNAd	GGCCCGAGAACGTATTCACC	288
	16SrRNArc	CGAGCGCAACCCTTGTTATC	
	16SrRNAd2	GTTCCAGTGTGACCGTTCAC	295
	16SrRNArc2	CTTAGAACTAACGCTGGCAG	
23S rRNA	23SrRNAd	CCTCTTAACCTTCCAGCACC	248
	23SrRNArc	GGTTAGGCTATAAGGGACCG	
tRNA^Ile^	tRNA^Ile^d	GATCATAGCTCAGGTGGTTAG	69
	tRNA^Ile^rc	GACCAGGATGAGTTGAACATC	
tRNA^Ala^	tRNA^Ala^d	GTTAAGGGACTCGAACCCTTG	65
	tRNA^Ala^rc	GTTTAGCTCAGTTGGCTAGAG	
*flaB*	flaBd	TCATTGCCATTGCAGATTGTG	278
	flaBrc	ACCTTCTCAAGGCGGAGTTAA	
16S rRNA - tRNA^Ala^	16SrRNArc	CGAGCGCAACCCTTGTTATC	683
	tRNA^Ala^d	GTTAAGGGACTCGAACCCTTG	
tRNA^Ala ^- tRNA^Ile^	tRNA^Ala^rc	GTTTAGCTCAGTTGGCTAGAG	781
	tRNA^Ile^d	GATCATAGCTCAGGTGGTTAG	
tRNA^Ile ^- 23S rRNA	tRNA^Ile^rc	GACCAGGATGAGTTGAACATC	2522
	23SrRNA3'd2	CTTATTACAGACTAAGCCTAAACGTC	
23S rRNA - 5S rRNA	23SrRNArc	GGTTAGGCTATAAGGGACCG	1403
	5SrRNAd	CCCTGGCAATAACCTACTC	
5S rRNA - 23S rRNA	5SrRNArc	CCCTGGTGGTTAAAGAAAAG	631
	23SrRNA3'd2	CTTATTACAGACTAAGCCTAAACGTC	

### Growth rate of *B. burgdorferi *and synthesis of DNA, RNA and protein under different conditions of nutrition and temperature

To identify the effect of growth rate and (p)ppGpp levels in *B. burgdorferi*, we examined growth and accumulation of DNA, RNA and protein in *B. burgdorferi *cultured at 34°C in BSK-H in the presence or absence of rabbit serum (an attempt at nutritional variation) and in *B. burgdorferi *cultured in BSK-H in the presence of rabbit serum at 34°C and 23°C (temperature variation). *B. burgdorferi *B31 was used for these experiments because the high cellular concentrations it reaches during *in vitro *culture (> 3 ×10^8 ^cells/ml) facilitated obtaining sufficient quantities of cells to permit measurement of DNA, RNA and protein by colorimetric assays [22,23]. Because rRNA constitutes more than 80% of total cellular RNA [11], rRNA was estimated from measurements of total RNA.

**Figure 3:**
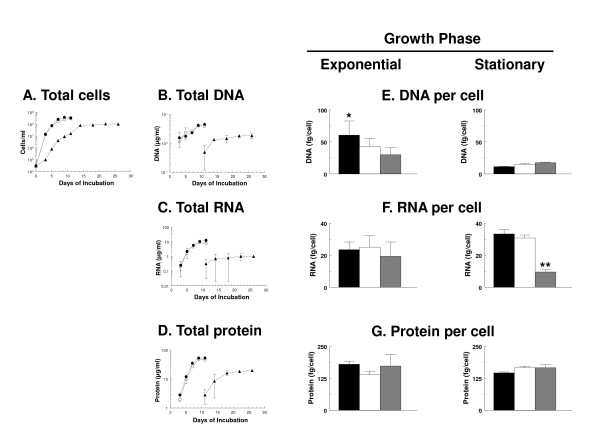
**Cell growth (A), total DNA (B), total RNA (C) and total protein (D) (mean ± SE) per ml in *B. burgdorferi *B31 cultured in BSK-H at 34°C in the presence (solid circle) or absence (open circle) of 6% rabbit serum, and in BSK-H at 23°C in the presence of 6% of rabbit serum (triangle); Data point symbols obscure the error bars in some cases; Mean (± SE) DNA (E), RNA (F) and protein (G) per *B. burgdorferi *B31 cell after culture in BSK-H containing 6% of rabbit serum at 34°C (black bar), in BSK-H containing no serum at 34°C (white bar), or in BSK-H containing 6% of rabbit serum at 23°C (gray bar).** Data from pooled values from washed exponentially growing cells (days 3-5 for the 34°C condition and day 11 for the 23°C condition) or washed stationary cells (days 7-11 for the 34°C condition and days 14-26 for the 23°C condition), and represent means of 4-8 values for DNA, RNA and protein from cells in exponential phase, and 12-18 values for cells in stationary phase. Data were analyzed by one-way analysis of variance with a Tukey-Kramer multiple comparisons post-test. *, DNA per cell was significantly greater (P < 0.05) in exponentially growing *B. burgdorferi *B31 cultured at 34°C in the presence of 6% rabbit serum than in stationary phase *B. burgdorferi *under any culture condition examined. **, RNA per cell was significantly lower (P < 0.05) in stationary phase *B. burgdorferi *B31 cultured at 23°C in the presence of 6% rabbit serum than in *B. burgdorferi *cultured at 34°C in the presence or absence of 6% rabbit serum.

**Figure 4:**
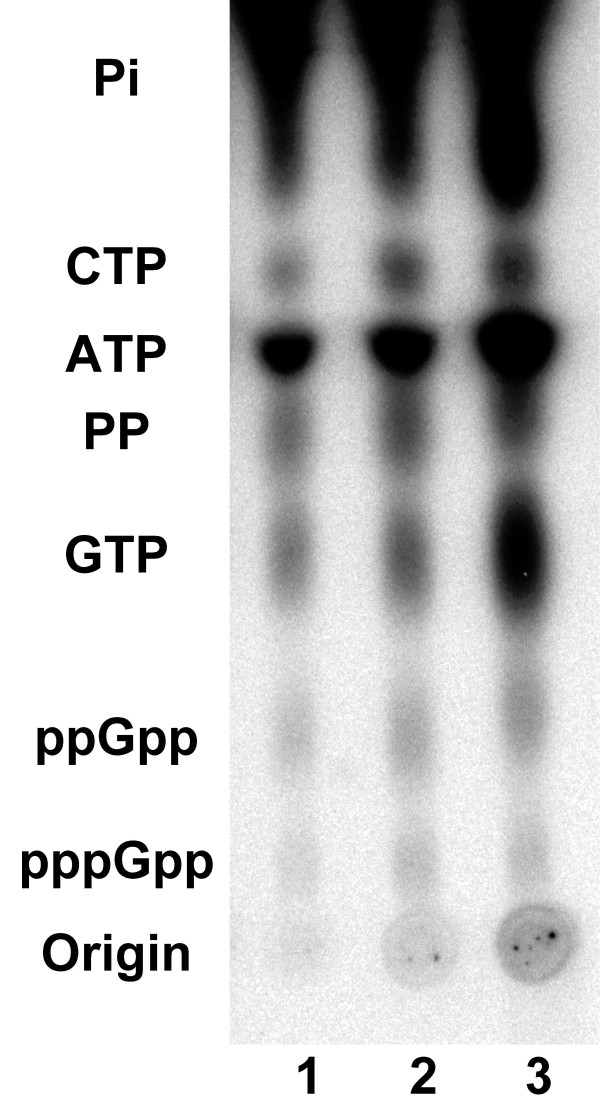
**Detection of (p)ppGpp in *B. burgdorferi *B31 grown at 34°C in BSK-H in the presence (lane 1) or absence (lane 2) of 6% rabbit serum, or in BSK-H at 23°C in the presence of 6% rabbit serum (lane 3)**. Similar amounts of (p)ppGpp were detected in cells under all three culture conditions.

At 34°C, the growth rate of *B. burgdorferi *and synthesis of total DNA, RNA and protein were unaffected by the presence or absence of rabbit serum as spirochetes grew from 3 × 10^4 ^to 3 × 10^8 ^cells/ml (Figure 3). Levels of RNA and protein per cell in *B. burgdorferi *were similar to those in slow-growing *E. coli *[8], while the level of DNA per cell was similar to that of normally dividing *E. coli *[8]. At 23°C, there was a lag in increases in *B. burgdorferi *cell numbers and total DNA, RNA and protein; in addition growth rate was slower, final concentrations of cells were three times lower (Figure 3A), as were total DNA, RNA and protein relative to those at 34°C (Figure 3B-D). These differences did not appear to be due to triggering of the stringent response by these environmental variations, since similar amounts of (p)ppGpp were detected in *B. burgdorferi *B31 grown in BSK-H in the presence or absence of rabbit serum at 34°C or in the presence of rabbit serum at 23°C (Figure 4). These results indicate that the absence of rabbit serum in BSK-H did not trigger slow growth at 34°C or changes in (p)ppGpp levels at either temperature.

For calculation of DNA, RNA and protein on a per cell basis, data from washed exponential and stationary phase cells were analyzed separately (see legend to Figure 3 for details). Since we could not obtain sufficient amounts of material for analysis of exponential growth at 23°C because of the relative insensitivity of colorimetric assays and high costs of large volumes of BSK-H culture medium, only the data from day 11 were used for this condition. At 34°C, there was significantly more DNA per cell in exponentially growing *B. burgdorferi *B31 cultures containing rabbit serum than at any of the other growth conditions (P < 0.05, one-way analysis of variance, Tukey-Kramer multiple comparison post-test) (Figure 3E). There was significantly less RNA per cell in stationary phase *B. burgdorferi *at 23°C than at 34°C (Figure 3F) (P < 0.05, one-way analysis of variance, Tukey-Kramer multiple comparison post-test). There was no significant difference in protein per cell under any growth condition at any temperature (Figure 3G). Because precise correlation between corresponding points on growth curves for cultures growing at different rates is difficult, it was therefore still unclear whether rRNA levels were regulated by growth rate or growth phase in *B. burgdorferi *B31.

### Effect of growth rate and stringent response on accumulation of 16S and 23S rRNA in *B. burgdorferi*

**Figure 5:**
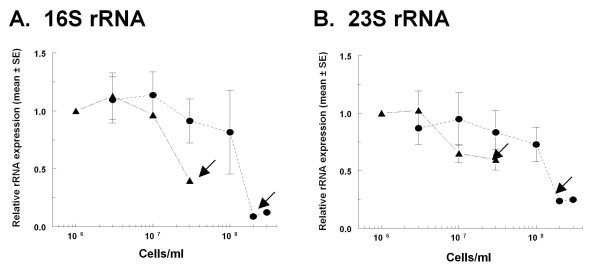
**Expression of 16S and 23S rRNA (mean ± SE) normalized to *flaB *mRNA in *B. burgdorferi *B31 grown in complete BSK-H at 34°C (solid circle) or at 23°C (triangle)**. Data are presented relative to normalized rRNA expression in 10^6 ^cells/ml of *B. burgdorferi *grown at 23°C in complete BSK-H for each rRNA species separately. See Materials and Methods for details. Arrows indicate the onset of stationary phase.

The apparent effect of growth rate on rRNA synthesis could be influenced by sampling *B. burgdorferi *that were in different growth phases at the two temperatures. Direct analysis of 16S and 23S rRNA levels in *B. burgdorferi *B31 grown in BSK-H containing serum at 34°C and 23°C revealed no difference in the levels of normalized 16S rRNA expression in cells grown at different temperatures when the cells were at similar points in the growth phase (Figure 5A). Examination of rRNA in the 23°C culture was begun at lower cell concentrations since this culture reached stationary phase at about 3-fold lower cell densities than cultures grown at 34°C (Figure 3A). Even though the average doubling time for *B. burgdorferi *B31 was 5 h at 34°C and 15 h at 23°C (Figure 3A), rRNA levels decreased significantly under both culture conditions with entry into stationary phase (P < 0.05, one-way analysis of variance, Tukey-Kramer multiple comparison post-test). A similar result was observed with 23S rRNA (Figure 5B). These results indicate that the apparent down-regulation of total RNA per cell in cultures grown at 23°C compared to cultures grown at 34°C (Figures 3C, F, 5AB) was in fact due to comparing cells that had spent a longer time in stationary phase at 23°C than those growing at 34°C, and was not the result of the decreased growth rate at the lower temperature.

**Figure 6:**
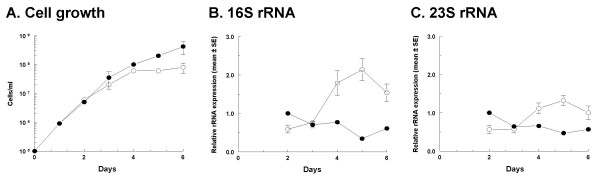
**Cell growth (A) and expression of 16S (B) and 23S (C) rRNA (mean ± SE) normalized to *flaB *mRNA in wild-type (solid circle) and Δ*rel_Bbu _*(open circle) *B. burgdorferi *297 grown in complete BSK-H at 34°C**. Data are presented relative to normalized rRNA expression at day two of wild-type cell culture as described in Materials and Methods.

To examine if the stringent response regulated rRNA levels in this bacterium, *B. burgdorferi *297 and its Δ*rel_Bbu _*derivative that could not synthesize (p)ppGpp were used [19]. Both strains multiplied at a similar rate in exponential phase in BSK-H at 34°C (Figure 6A) but the deletion mutant stopped dividing after day four of culture while densities of the wild-type strain continued to increase (Figure 6A). In wild-type *B. burgdorferi*, 16S and 23S rRNA levels were very similar at 2 to 4 days of culture and decreased only slightly toward the end of the growth curve when the culture was reaching its maximum density and increased its doubling time (Figures 6B, C). In contrast, rRNA levels in *B. burgdorferi *Δ*rel_Bbu _*peaked at day five for both rRNA species, the first day of culture when cell densities of Δ*rel_Bbu _*did not increase (Figure 6). The reverse correlation between cell division and rRNA accumulation in *B. burgdorferi *Δ*rel_Bbu _*strongly suggests that *rel_Bbu _*is necessary for stringent control of rRNA synthesis in *B. burgdorferi*. This accumulation of rRNA is reminiscent of what occurs in the relaxed phenotype of *E. coli relA *mutants [9,24,25].

## Discussion

We have demonstrated the existence of three different transcripts from the DNA region of *B. burgdorferi *coding for ribosomal RNA. One transcript contained 16S rRNA and tRNA^Ala^, a second contained only tRNA^Ile^, and a third contained both sets of 23S-5S rRNA genes in tandem. These results confirm previous predictions that *B. burgdorferi *rRNA genes were not transcribed as a single unit [15,16]. *B. burgdorferi *is not the only spirochete in which rRNA genes are not organized into operons containing 16S-23S-5S genes in tandem [26]. The *B. garinii *genome encodes one copy of 16S and two copies each of 23S and 5S rRNA genes organized similarly to those of *B. burgdorferi *[27], while *B. japonica *IKA2 has only a single copy of the 23S-5S rRNA gene [28]. Other spirochetes also have a limited number of rRNA genes which are often not organized in operons containing 16S-23S-5S genes in tandem. An early report indicated that the spirochete *Leptospira interrogans *had two copies of 16S and single copies of 5S and 23S rRNA genes located far from each other and most probably not expressed together [29]. More recent whole genome sequencing has shown that the number of rRNA genes differs between two *L. interrogans *serovars. *L. interrogans *sv. Copenhageni has two copies of 23S, two copies of 16S, and one copy of 5S rRNA genes, while *L. interrogans *sv. Lai has one copy of 23S rRNA, two copies of 16S rRNA, and one copy of 5S rRNA genes [30,31]. The rRNA genes of both *L. interrogans *serovars are physically separated from each other and do not appear to form operons. However, not every spirochetal genome codes for individual rRNA genes that are not organized into operons. *Treponema pallidum *and *T. denticola *have two operons each coding for one copy of 16S, 23S and 5S rRNA [32,33]. This variation in copy number and location of rRNA genes suggests that rRNA synthesis is controlled differently in different spirochetes.

It has been assumed that the presence of multiple copies of transcriptional units of rRNA in the order 16S, 23S and 5S rRNA facilitates the adaptation of bacteria to conditions that rapidly change their growth rate because they permit rapid changes in ribosomal synthesis [11,14,26]. In *E. coli*, sequential deletion of rRNA genes is accompanied by a decrease in the ability of the mutants to accelerate their growth rate under changing media conditions [34]. The location of rRNA genes close to the origin of replication in *E. coli *insures parallelism between replication and rRNA gene transcription and results in their high gene dosage in rapidly replicating cells [34]. That slow-growing bacteria such as spirochetes, mycoplasma and mycobacteria have a reduced number of rRNA gene copies could be intuitively related to a decreased adaptability resulting from their low numbers of rRNA copies and to a lack of coordinate transcription of the three RNA populations and DNA replication [35,36]. We have previously shown that inactivation of one of the 23S RNA genes in *B. burgdorferi *does not have any apparent effect on its adaptability to different growth conditions [37]. Moreover, a similar experiment has been performed in nature because *B. japonica *strain IKA2 lacks a second copy of the 23S-5S rRNA gene cluster without any apparent adverse effects [28]. This suggests that *B. burgdorferi *has already adapted its growth rate to that permitted by its reduced number of rRNA genes. It thus appears that ascertainment of the biological role of differences in rRNA gene copy number in various bacterial species will require an extensive comparative analysis of the adaptability of bacteria with high and low numbers of rRNA genes to different growth conditions before any clear cut conclusions can be drawn.

Two major mechanisms regulating rRNA synthesis in *E. coli *are growth rate and the stringent response [9,11]. Our efforts to determine if *B. burgdorferi *rRNA synthesis was controlled by growth rate at a single temperature have been repeatedly frustrated by the still undefined nutritional requirements of *B. burgdorferi *and the lack of simple culture media for this organism [38,39]. We previously reported that (p)ppGpp levels in *B. burgdorferi *grown in BSK did not vary despite 10-fold reductions of yeastolate, neopeptone or rabbit serum [18]. We have now found that complete omission of rabbit serum from BSK-H did not affect growth of *B. burgdorferi *B31 at 34°C (Figure 3) or (p)ppGpp levels at 34°C or 23°C (Figure 4). It was thus not possible to alter *B. burgdorferi *growth rate at a given temperature by changing the composition of its culture medium [11,40].

The slower growth of *B. burgdorferi *B31 at 23°C compared to 34°C correlated well with slower accumulation of total DNA, RNA and protein. Although there was a lag in cell number, total DNA and total protein in cells grown at the lower temperature, the amounts of DNA and protein per cell were similar at both temperatures. As expected, the amount of DNA per rapidly dividing exponential phase cells was higher than in more slowly dividing stationary phase cells. The slower rate of increase in total RNA in stationary phase cultures at the lower temperature also resulted in a significant difference in RNA per cell under these two conditions. Although these results were in agreement with the hypothesis that growth rate regulates rRNA synthesis in *B. burgdorferi*, further investigation determined that growth *phase *and not growth *rate *regulated rRNA levels under these conditions (Figure 5). Importantly, even though *B. burgdorferi *was grown for up to 11 days in 34°C culture and for 28 days in 23°C culture in our experiments, about 80% of all cells at this stationary phase stage are viable (R. Iyer and I. Schwartz, unpublished results), and non-viability cannot therefore account for the large decrease in rRNA levels in stationary phase in these cultures.

Amounts of 16S and 23S rRNA that were first normalized to mRNA amounts for constitutively expressed *flaB *and then additionally normalized to levels at 23°C and 10^6 ^cells/ml were similar in rapidly growing (34°C) and slowly growing (23°C) cultures when compared at the same growth phase; both RNA species decreased as the cultures progressed toward stationary phase (Figure 5). Production of constant levels of rRNA in exponentially growing *B. burgdorferi *that were independent of bacterial doubling time and the down-regulation of rRNA during stationary phase is similar to results obtained with *Salmonella enterica *sv. Typhimurium cultured in the same medium at different temperatures [40]. While cellular contents of DNA, RNA, and protein in cultures of *S. *Typhimurium grown in media of different nutritional content at a given temperature depended on growth rate, DNA, RNA, and protein per cell were nearly constant in cultures grown in the same medium at different temperatures and did not depend on growth rate [40].

We have previously shown that (p)ppGpp is necessary for the transition between exponential and stationary phase in *B. burgdorferi *[19], suggesting that rRNA synthesis may not be totally independent of (p)ppGpp, and that rRNA levels may be determined by interplay between two factors in this organism, growth phase and (p)ppGpp levels. In the present study, we found that both *B. burgdorferi *rRNA operons were misregulated in the absence of (p)ppGpp, and failed to down-regulate 16S and 23S rRNA levels during the transition to the stationary phase. Although our previous experiments with tick cell cultures suggested that growth-related mechanisms other than (p)ppGpp modulated rRNA synthesis in *B. burgdorferi *[17,18], it is evident that the stringent response is also important for regulation of rRNA synthesis.

The mechanism by which (p)ppGpp regulates rRNA synthesis in *B. burgdorferi *during the transition phase and what other factors might be involved in this regulation is not yet clear. The accumulation of rRNA in *B. burgdorferi *Δ*rel_Bbu _*suggests that this mutant behaves similarly to a relaxed phenotype *relA *mutant of *E. coli *(Figures 6B, C) [9,24,25]. This unbalanced growth may be responsible for the lack of cell division of the *B. burgdorferi *Δ*rel_Bbu _*mutant in the stationary phase of growth (Figure 6A). *B. burgdorferi *has no homolog to the transcription factor DksA that acts as a cofactor in the repression of rRNA genes by (p)ppGpp in *E. coli *[10,41,42]. Even though *B. burgdorferi *codes for a homolog to the GTP-binding protein gene *cgtA *(BB0781) [10], this GTPase regulates (p)ppGpp levels only during exponential growth and does not have an effect during the stringent response [43]. Although not fully characterized, the role of the stringent response in the regulation of rRNA levels during stationary phase might have an effect on the ability of *B. burgdorferi *to survive in flat ticks or persist in animals. This might be accomplished perhaps by slowing down protein synthesis and conserving resources until nutritional conditions improve [44-46].

## Conclusions

We have confirmed the prediction that *B. burgdorferi *rRNA genes are transcribed into three separate transcripts. We have also found that differences in expression of the rRNA operons associated with *B. burgdorferi *growth at different temperatures is regulated by growth phase rather than by growth rate, and that *rel_Bbu _*and its product (p)ppGpp is required for this regulation. These findings suggest that this bacterium has mechanisms for coordinated regulation of rRNA gene synthesis perhaps in response to metabolic changes triggered by entry into the stationary phase. Identification of these mechanisms is likely to be relevant to understanding the ability of *B. burgdorferi *to persist in the tick vector and the mammalian host.

## Methods

### Bacterial strains and growth conditions

Infectious, low-passage *B. burgdorferi *N40 was provided by Dr. L. Bockenstedt (Yale University, New Haven, CT). Non-infectious high-passage *B. burgdorferi *B31 was provided by Dr. J. Radolf (University of Connecticut Health Center, Farmington, CT). *B. burgdorferi *297 (clone BbAH130) was provided by Dr. M. Norgard (University of Texas Southwestern Medical Center, Dallas, TX). This infectious wild-type strain was the parental strain for the Δ*rel_Bbu _B. burgdorferi *[19]. *B. burgdorferi *strains were maintained at 34°C in BSK-H (Sigma Chemical Co., St. Louis, MO) supplemented with 6% rabbit serum (Sigma) (complete BSK-H) if not otherwise stated. Cell numbers were determined by dark-field microscopy as previously described [17].

### DNA isolation and PCR

DNA from *B. burgdorferi *was isolated using High Pure PCR Template Preparation Kit (Roche Diagnostics Corporation, Indianapolis, IN). PCR amplification was performed using *Taq *DNA polymerase (Sibgene, Derwood, MD). Primers used for PCR are listed in Table 1. PCR was performed in a final volume of 10 μl using an Idaho Technology RapidCycler (Idaho Technology Inc., Salt Lake City, UT). The amplification program consisted of denaturation at 94°C for 15 sec; followed by 37 cycles of 94°C for 10 sec-53°C for 10 sec-72°C for 50 sec (for tRNA^Ala^-tRNA^Ile ^region) or for 2 min (for tRNA^Ile^-23S rRNA region); and final extension at 72°C for 30 sec.

### RNA isolation and RT-PCR

RNA from *B. burgdorferi *was isolated with TRIzol Reagent (Invitrogen Life technology, Carlsbad, CA.) according to the manufacturer's recommendations and was treated with RQ1 RNase-free DNase (Promega Corporation, Madison, WI) to eliminate DNA contamination. Primers used for RT-PCR are listed in Table 1 and their location shown in Figure 1. RT-PCR was performed using the Access RT-PCR system (Promega) in the RapidCycler using the following conditions: reverse transcription at 48°C for 45 min, denaturation at 94°C for 2 min; followed by 35 cycles of 94°C for 10 sec-52°C (5S rRNA, tRNA^Ile^, tRNA^Ala^, tRNA^Ala ^- tRNA^Ile^, tRNA^Ile ^- 23S rRNA, 23S rRNA - 5S rRNA and 5S rRNA - 23S rRNA intergenic regions) or 56°C (16S rRNA, 23S rRNA and 16S rRNA-tRNA^Ala ^intergenic region) for 10 sec-68°C for 50 sec (all rRNA and tRNA genes and their intergenic regions except tRNA^Ile^-23S rRNA and 23S rRNA- 5S rRNA intergenic regions) or for 2 min (tRNA^Ile^-23S rRNA and 23S rRNA-5S rRNA intergenic regions); and final extension at 68°C for 5 min.

### Isolation and measurement of total DNA and total RNA

*B. burgdorferi *B31 were grown from 3 × 10^4 ^cells/ml in BSK-H with or without 6% rabbit serum at 34°C, or in BSK-H with 6% of rabbit serum at 23°C. *B. burgdorferi *from 50-70 ml cultures were collected by centrifugation, washed twice with PBS, pH 7.5, resuspended in 900 μl of PBS and mixed with 100 μl of 50% trichloroacetic acid at 0°C. After at least 15 min at 0°C, the cells were collected on glass fiber filters without binders (Millipore, Ireland, 25 mm diameter, 2.7 μm particle penetration) and washed with 20 ml of 5% trichloroacetic acid. Filters containing the entrapped cells were folded, placed in the bottom of a test tube (13 × 100 mm) and covered with 2 ml of 5% trichloroacetic acid. The tubes were capped and placed in a 90°-95°C water bath for 20 min. After cooling, glass filters were sedimented by centrifugation and DNA and RNA concentrations were determined colorimetrically on aliquots of the supernatant fluid by diphenylamine (for DNA) or orcinol (for RNA) assays [22,23]. Each experiment was repeated twice with two technical replicates. Data are presented as means ± SE.

### Measurement of total protein

*B. burgdorferi *B31 were grown as above. *B. burgdorferi *cells from 1.5 ml cultures were collected by centrifugation, washed twice with PBS, pH 7.5, to remove any adherent proteins derived from the culture medium, resuspended in 50 μl of lysis buffer containing 50 mM Tris-HCl, pH 7.5; 0.15 M NaCl; 1 mM EDTA; 0.1% Triton X-100 and incubated on ice for 10 minutes. Total protein was measured using the Bradford method [47] (Bio-Rad Protein Assay, Bio-Rad Laboratories) with a bovine serum albumin standard. Each experiment was repeated twice with two technical replicates. Data are presented as means ± SE.

### Detection of (p)ppGpp

(p)ppGpp was extracted from [^32^P]-labeled *B. burgdorferi *and chromatographed on cellulose PEI-TLC plates (Selecto Scientific, Suwanee, GA) as previously described [17]. Plates were air-dried, exposed to phosphor screen (Molecular Dynamics, Sunnyvale, CA) for 12 to 24 h and scanned using a Storm 860 PhosphorImager (Molecular Dynamics).

### Reverse transcription and Real-time PCR

cDNA synthesis was performed with 1 μg of total *B. burgdorferi *RNA using random primers p(dN)_6 _(Roche) and avian myeloblastosis virus reverse transcriptase (Promega) according to the manufacturer's recommendations. To quantify *flaB *mRNA and 16S and 23S rRNA, the resulting cDNAs were amplified and analyzed on a LightCycler Real-time PCR instrument (Roche) using LightCycler Master SYBR Green I Mixture (Roche). PCR was performed in glass capillaries in a final volume of 20 μl as previously described [18]. The amplification program consisted of denaturation at 95°C for 2 min; followed by 35 cycles of 95°C for 1s-55°C (*flaB *and 23S rRNA) or 57°C (16S rRNA) for 5 s-72°C for 10 s. PCR reactions were performed at least twice for each RNA isolate. RNA isolated from at least two independent cultures was used for experiments with temperature change. PCR reactions were performed 4 times for each RNA isolate in experiments with *rel_Bbu _*mutant, and RNA was isolated from one culture for days 2, 4 and 5, and from two independent cultures for days 3 and 6. Results using two different primer sets for 16S rRNA quantification (Table 1, Figure 1) were similar and were therefore combined. Genomic DNA from 10^3^-10^6 ^cells of the corresponding *B. burgdorferi *strain was used as a standard to estimate the amount of cDNA for genes studied in each Real-time PCR. Samples were normalized to the amount of cDNA of constitutively expressed *flaB*. Relative rRNA expression levels (copies rRNA/copies *flaB*) were computed for each individual rRNA species (16S or 23S rRNA). Because *flaB *mRNA expression is constitutive [48,49], and *flaB *is located on the chromosome distal to the origin of replication [50] which ensures that there is only one copy of *flaB*/borrelial cell, normalization with *flaB *is adequate. In RT RT-PCR experiments with different temperature, these expression levels were further normalized to expression during growth in BSK-H at 23°C and 10^6 ^cells/ml. In experiments with Δ*rel_Bbu_*, the expression levels were normalized to expression of wild-type at day two - the first day when RNA was collected, separately for 16S and 23S rRNA. Relative rRNA expression of each rRNA species is presented as mean ± SE.

### Statistical methods

Differences in mean levels of rRNA transcription, cell numbers and amounts of total DNA, RNA and protein were statistically analyzed using a one-way analysis of variance with a Tukey-Kramer multiple comparisons post-test. Differences were deemed significant if P < 0.05.

## List of Abbreviations

BSK: Barbour-Stoenner-Kelly; cDNA: DNA complementary to RNA; PCR: polymerase chain reaction; (p)ppGpp: (guanosine-3'-dipyrophosphate-5'-triphosphate and guanosine-3',5'-bispyrophosphate, collectively); rRNA: ribosomal RNA; tRNA: transfer RNA.

## Authors' contributions

JVB carried out the molecular genetic and growth studies and drafted the manuscript. HPG performed the statistical analysis, participated in the coordination of the study and helped draft the manuscript. IS participated in the design of the study and helped draft the manuscript. FCC conceived of the study, participated in its design and coordination and helped draft the manuscript. All authors read and approved the final manuscript.

## Authors' information

JVB is currently at the Department of Microbiology and Immunology, Emory University School of Medicine, 1510 Clifton road, Atlanta, GA 30322, USA. HPG is currently at the Department of Pathology, Basic Science Building, New York Medical College, Valhalla, NY 10595, USA. IS and FCC are currently at the Department of Microbiology and Immunology, Basic Science Building, New York Medical College, Valhalla, NY 10595, USA.
